# Examining the Associations among Fibrocystic Breast Change, Total Lean Mass, and Percent Body Fat

**DOI:** 10.1038/s41598-018-27546-3

**Published:** 2018-06-15

**Authors:** Yuan-Yuei Chen, Wen-Hui Fang, Chung-Ching Wang, Tung-Wei Kao, Yaw-Wen Chang, Hui-Fang Yang, Chen-Jung Wu, Yu-Shan Sun, Wei-Liang Chen

**Affiliations:** 1 Department of Internal Medicine, Tri-Service General Hospital Songshan Branch, Tri-Service General Hospital; and School of Medicine, National Defense Medical Center, Taipei, Taiwan Republic of China; 2 Division of Family Medicine, Department of Family and Community Medicine, Tri-Service General Hospital; and School of Medicine, National Defense Medical Center, Taipei, Taiwan Republic of China; 3 Division of Geriatric Medicine, Department of Family and Community Medicine, Tri-Service General Hospital; and School of Medicine, National Defense Medical Center, Taipei, Taiwan Republic of China; 4 Graduate Institute of Clinical Medical, College of Medicine, National Taiwan University, Taipei, Taiwan Republic of China; 5 Division of Family Medicine, Department of Community Medicine, Taoyuan Armed Forces General Hospital, Taoyuan, Taiwan Republic of China

## Abstract

Fibrocystic breast change (FBC) is extremely common and occurrs in 90% of women during their lives. The association between body composition and risk of breast cancer is well established. We hypothesized that the effect might exist during the development of FBC. Our aim was to examine the relationships of total lean mass (TLM) and percent body fat (PBF) with FBC in a general female population. In total, 8477 female subjects aged 20 years or older were enrolled in the study at the Tri-Service General Hospital in Taiwan from 2011 to 2016. Comprehensive examinations including biochemical data, measurements of body composition and breast ultrasound were performed. PBF was positively associated with the presence of FBC (OR = 1.039, 95%CI: 1.018–1.060), and TLM showed the opposite result (OR = 0.893, 95%CI: 0.861–0.926). Condition of metabolic syndrome (MetS), diabetes (DM) and fatty liver modified the association between PBF and FBC (P < 0.001, P = 0.032 and P = 0.007, respectively). Female subjects diagnosed with MetS, DM, and fatty liver had higher risk of developing FBC than control subjects (OR = 1.110, 95%CI: 1.052–1.171; OR = 1.144, 95%CI: 1.024–1.278; OR = 1.049, 95%CI: 1.019, 1.080). Those with higher PBF (for highest quartile versus lowest, OR = 2.451, 95%CI: 1.523–3.944) or lower TLM (for highest quartile versus lowest, OR = 0.279, 95%CI: 0.171–0.455) had increased risk of developing FBC. In conclusion, increased PBF and reduced TLM were likely to predict the risk of the presence of FBC in a general female population.

## Introduction

Fibrocystic breast change (FBC), also termed **“**fibrocystic breast disease”, is the general, all-inclusive term, for a whole range of common and benign breast disorders^1^. FBC is most common among premenopausal women aged 20–50 years old^2^. Such changes comprise all types of benign conditions such as cysts, papilloma, apocrine metaplasia, epithelial hyperplasia, and adenosis^3^. Several studies indicated that the lifetime prevalence of FBC in women might be as high as 70% to 90%^4,5^.

These fibrocystic changes might be discovered incidentally by radiologists in women who began screening. Generally, FBC is not related to breast cancer. However, accumulating evidence has proposed that the breast cancer risk is involved with FBC. Women with nonproliferative lesions did not have a risk for breast cancer, whereas those with proliferative lesions without atypia had an approximately 2-fold increase in risk and those with atypical ductal or lobular hyperplasia had an approximately 5-fold increase in risk^6,7^. In a study composed of women diagnosed with benign breast disease, females with atypical epithelial proliferation had higher risk than women over 55 years with the same diagnosis^8^.

The associations between body composition and breast cancer have been examined and well established for decades^9,10^. Adipose tissue might increase the risk of breast cancer by influencing sex hormone balance, endocrine function and adipokine expression^11^. Recently, lean body mass was found to represent a powerful endocrine, immune and hormonal influence within the body^12^. However, few studies have addressed the effect of obesity on FBC and the association was largely unknown.

Due to the potential risk of FBC in the development of breast cancer, we were inspired to examine whether the alteration in body composition would increase the risk of FBC. The aim of our study was to investigate the associations between FBC with percent body fat (PBF) and total lean mass (TLM) with FBC in a large-scale general female population who had undergone health examinations in a medical center in Taiwan.

## Results

### Characteristics of the study sample with or without the presence of FBC

The demographic characteristics including age, body composition indices and laboratory biochemical data of participants with or without FBC are represented in Table 1. The mean age of those with and without FBC were 45.77 ± 11.81 and 42.96 ± 16.19 years old. TLM and PBF in subjects with FBC were 20.79 ± 2.68 kg and 32.17 ± 6.67%, respectively, and in those without FBC were 20.74 ± 3.08 kg and 31.91 ± 6.66% respectively.

**Table 1:** Characteristics of study sample with or without fibrocystic breast change.

Variables	Fibrocystic change (−) (N = 7478)	Fibrocystic change (+) (N = 999)	P Value
**Continuous Variables, mean (SD)**			
Total lean mass (kg)	20.74 (3.08)	20.79 (2.68)	0.690
PBF (%)	31.91 (6.66)	32.17 (6.67)	0.370
Age (years)	42.96 (16.19)	45.77 (11.81)	<0.001
BMI (kg/m^2^)	22.94 (4.11)	22.76 (3.66)	0.211
Total cholesterol (mg/dL)	185.50 (35.53)	190.19 (34.50)	<0.001
Uric acid (mg/dL)	4.74 (1.10)	4.70 (1.03)	0.293
Creatinine (mg/dL)	0.68 (0.21)	0.67 (0.11)	0.154
AST (U/L)	18.97 (13.54)	19.57 (14.75)	0.208
Albumin (g/dL)	4.43 (0.29)	4.43 (0.27)	0.395
hsCRP (mg/dL)	0.21 (0.43)	0.19 (0.27)	0.234
TSH (IU/mL)	2.38 (1.85)	2.45 (2.07)	0.366
**Category Variables, (%)**			
Proteinuria	6996 (29.0)	209 (26.7)	0.147
Smoking	650 (9.1)	62 (7.7)	0.178
Drinking	1659 (28.3)	202 (28.9)	0.752

### Associations between the presence of FBC with TLM and PBF

As shown in Table 2, the relationships between different anthropometric indices and the presence of FBC were analyzed by logistic regression. TLM showed an inverse tendency for prediction of the risk of FBC with ORs of 0.811, 0.884 and 0.893 (95%CI = 0.789–0.833, 0.853–0.916, 0.861–0.926) in Model 1, 2 and 3, respectively. On the other hand, PBF was positively associated with the presence of FBC with ORs of 1.082, 1.045 and 1.039 (95%CI = 1.066–1.099, 1.025–1.066, 1.018–1.060) in Model 1, 2 and 3, respectively.

**Table 2:** Association between different anthropometric parameters and the presence of fibrocystic breast change.

Models	Model^a^ 1 OR (95% CI)	*P* Value	Model^a^ 2 OR (95% CI)	*P* Value	Model^a^ 3 OR (95% CI)	*P* Value
Variables	Fibrocystic breast change					
Total lean mass	0.811 (0.789, 0.833)	<0.001	0.884 (0.853, 0.916)	<0.001	0.893 (0.861, 0.926)	<0.001
PBF	1.082 (1.066, 1.099)	<0.001	1.045 (1.025, 1.066)	<0.001	1.039 (1.018, 1.060)	<0.001

^a^Adjusted covariates:

Model 1 = unadjusted.

Model 2 = Model 1 + age, proteinuria, serum total cholesterol, uric acid, creatinine, AST, albumin, hsCRP.

Model 3 = Model 2 + history of smoking, drinking.

### Association between PBF and the presence of FBC with or without different outcomes

The associations between PBF and diagnoses of FBC with or without underlying diseases such as metabolic syndrome (MetS), diabetes mellitus (DM) and fatty liver performed by multivariable logistic regression are listed in Table 3. Condition of MetS, DM and fatty liver modified the association between PBF and FBC (P for interaction <0.001, = 0.032 and = 0.007, respectively). People with or without MetS and DM all had a predictive ability for the presence of FBC with ORs of 1.110, 1.031 (95%CI = 1.052–1.171; 1.005–1.058) and 1.144, 1.037 (95%CI = 1.024–1.278; 1.016–1.059), respectively, in the fully adjusted model. However, no significant difference was noted for relationship between individuals without fatty liver and FBC. PBF in subjects who had fatty liver was positively correlated with the presence of FBC with ORs of 1.049 (95%CI = 1.019–1.080).

**Table 3:** Association between body fat percentage and fibrocystic breast change in the presence of different outcomes.

	Models	Model^a^ 1 OR (95% CI)	*P* Value	Model^a^ 2 OR (95% CI)	*P* Value	Model^a^ 3 OR (95% CI)	*P* Value
	**Variables**	Fibrocystic breast change					
PBF	**MetS** (−)	1.089 (1.067, 1.111)	<0.001	1.037 (1.011, 1.063)	0.005	1.031 (1.005, 1.058)	0.019
	**MetS** (+)	1.147 (1.102, 1.193)	<0.001	1.119 (1.063, 1.177)	<0.001	1.110 (1.052, 1.171)	<0.001
	**DM** (−)	1.084 (1.067, 1.101)	<0.001	1.043 (1.023, 1.064)	<0.001	1.037 (1.016, 1.059)	<0.001
	**DM** (+)	1.146 (1.051, 1.249)	0.002	1.143 (1.029, 1.270)	0.013	1.144 (1.024, 1.278)	0.017
	**Fatty liver** (−)	1.076 (1.051, 1.102)	<0.001	1.033 (1.003, 1.063)	0.033	1.027 (0.997, 1.058)	0.080
	**Fatty liver** (+)	1.110 (1.086, 1.134)	<0.001	1.058 (1.029, 1.088)	<0.001	1.049 (1.019, 1.080)	<0.001

^a^Adjusted covariates:

Model 1 = unadjusted.

Model 2 = Model 1 + age + BMI + proteinuria, serum total cholesterol, uric acid, creatinine, AST, albumin, hsCRP, TSH.

Model 3 = Model 2 + history of smoking, drinking.

### Association between anthropometric indices in quartiles with the presence of FBC

As shown in Table 4, quartile analysis was used for TLM and PBF to examine the dose-dependent effect on the association between anthropometric parameters and FBC. In line with our expectations, participants with reduced TLM had an increased risk of developing FBC with an OR of 0.279 (95%CI = 0.171–0.455) in the fully adjusted model. Those with elevated PBF had an increased risk of the presence of FBC with an OR of 2.451 (95%CI = 1.523–3.944) in the fully adjusted model.

**Table 4:** Association between anthropometric parameters in quartile with the presence of fibrocystic breast change.

Variable		Model^a^ 1 OR (95% CI)	*P* Value	Model^a^ 2 OR (95% CI)	*P* Value	Model^a^ 3 OR (95% CI)	*P* Value
	**Quartiles**	Fibrocystic breast change					
Total lean mass	**Q2 v.s. Q1**	0.857 (0.634, 1.158)	0.315	0.840 (0.613, 1.150)	0.276	0.845 (0.617, 1.158)	0.294
	**Q3 v.s. Q1**	1.002 (0.749, 1.341)	0.988	1.081 (0.782, 1.493)	0.638	1.112 (0.803, 1.539)	0.522
	**Q4 v.s. Q1**	0.084 (0.058, 0.122)	<0.001	0.235 (0.146, 0.379)	<0.001	0.279 (0.171, 0.455)	<0.001
PBF	**Q2 v.s. Q1**	2.167 (1.512, 3.104)	<0.001	1.494 (1.021, 2.188)	0.039	1.378 (0.939, 2.023)	0.101
	**Q3 v.s. Q1**	3.320 (2.336, 4.718)	<0.001	1.864 (1.248, 2.786)	0.002	1.610 (1.071, 2.420)	0.022
	**Q4 v.s. Q1**	4.794 (3.407, 6.748)	<0.001	2.935 (1.843, 4.673)	<0.001	2.451 (1.523, 3.944)	<0.001

^a^Adjusted covariates:

Model 1 = unadjusted.

Model 2 = Model 1 + age + BMI + proteinuria, serum total cholesterol, uric acid, creatinine, AST, albumin, hsCRP, TSH.

Model 3 = Model 2 + history of smoking, drinking.

Range of TLM in quartile.

Q1: <19.20; Q2: 19.20–21.20; Q3: 21.20–23.80; Q4: >23.80.

Range of PBF in quartile.

Q1: <25.90; Q2: 25.90–30.90; Q3: 30.90–35.60; Q4: >35.60.

## Discussion

In our study, we highlighted the important role of body composition in the process of FBC. Subjects with higher TLM had a lower risk of developing FBC. In contrast, higher PBF was significantly associated with an increased risk of the presence of FBC. It appeared that TLM played a protective role; however, increased PBF was detrimental to the general female population. To the best of our knowledge, the present study was the first to examine the relationship between different anthropometric parameters and FBC in a cross-sectional study composed of a large female general population.

The most significant contributing factor to FBC was the normal hormonal variation of women during the menstrual cycle^13^. Sex hormonal alterations with estrogen dominance over progesterone were considered to contribute to the development of hyperproliferation of breast tissue^13^. Adipose tissue has been suggested as an endocrine organ that secretes numerous hormones such as sex hormones^14^. Excessive body fat could raise levels of estrogen and increase the risk of hormone-receptor-positive breast cancer^15^. Numerous studies have reported that postmenopausal women placed on hormone replacement therapy had symptoms of FBC, indicating that hormones might play a role^16^. Aside from estrogen and progesterone, prolactin also led to FBC by expression outside of the breast and acting on the breast in important ways^17^. Prolactin was responsible for the growth and development of mammary glands^18^. There were specific prolactin receptors in breast tissue that increased during pregnancy and throughout estrogen therapy^19^. In a previous study, obesity and increased body fat were reported to be related to high levels of prolactin^20^. Kok *et al*. demonstrated that release of prolactin was enhanced in obese premenopausal women and was particularly associated with the amount of visceral fat^21^. The above evidence supported our findings that increased PBF was associated with a high risk of FBC in a general female population.

In a case-control study, a higher fat-muscle ratio was associated with increased risk of breast cancer, whereas muscle fraction was negatively associated^22^. The term “sarcopenic obesity”, known as TLM loss with fat tissue accumulation was common in breast cancer survivors^23^. TLM loss appears to be associated with metabolic abnormalities and is a positive predictor of adverse outcomes, such as chronic heart failure, chronic kidney disease and cancer cachexia^24–26^. Villasenor *et al*. reported that sarcopenia was associated with an increased risk of breast-cancer-specific mortality. It is important to improve prognosis by maintaining and increasing skeletal muscle mass^27^. The direct mechanism underlying the effect of lean body mass on breast diseases and cancer remains unknown. Concurrent lean mass loss caused by fat tissue accumulation might be a plausible explanation for the induction of elevated levels of estrogen in the development of FBC.

There were still potential limitations in the study. First, a cross-sectional design could not be assessible for casual inference between anthropometric indices and the presence of FBC. A longitudinal survey was suggested to be examined in further studies. Second, only Taiwanese females had enrolled in the study from health examinations at a single medical center. Limited ethnic diversity might not reflect the association in different ethnicities. Last, the measurement of body composition in the health check-up was performed by BIA, but not DEXA, a standard measurement for body composition with higher accuracy.

## Conclusion

Our findings highlighted the associations of TLM and PBF with the presence of FBC in a general female population. Decreased fat mass and increased lean mass might reduce the risk of FBC and even retard the progress of cancer. A better understanding of the pathophysiological underlying shared associations of TLM and PBF may provide biological insights into the etiology of FBC.

## Methods

### Study design

A total of 69226 participants aged 20 years and older were enrolled in health examinations at Tri-Service General Hospital from 2011 to 2016, and all characteristics of the study sample were analyzed in the retrospective cross-sectional study. Study approval was conduct by the Institutional Review Board (IRB) of Tri-Service General Hospital (TSGH), Taiwan. The TSGH IRB waived the need to obtain individual informed consent because these data were analyzed anonymously. Based on our inclusion criteria, males were excluded in the first step. Female participants with missing biochemical data and those lacking comprehensive examinations were excluded. In all, 8477 eligible subjects were included in the final analysis.

### Diagnosis of fibrocystic breast change

The study sample in our study was composed of a Taiwanese general female population. Several studies had reported that the morphological view of breast tissue in Asian women is denser than that in Caucasian women^28^. The breast tissue was dense and tightly packed with lobules, ducts and connective tissue in young females^29^. Due to the above supportive evidence, breast ultrasound was better than mammography for evaluating breast condition. Ultrasound imaging used sound waves to produce pictures of the internal structures of the breast. The radiographic features of breast ultrasound for FBC showed prominent fibroglandular tissue in palpable nodules without discernible mass or small cysts in the mammary zone^30^.

### Data collection

The baseline data in the present study included age, body composition [body mass index (BMI), TLM, and PBF], laboratory data [serum total cholesterol (TC), uric acid (UA), creatinine (Cr), aspartate aminotransferase (AST), albumin, highly sensitive C-reactive protein (hsCRP), and thyroid-stimulating hormone (TSH)], and personal history (proteinuria, cigarette smoking, alcoholic consumption). A self-reported questionnaire was used to collect age, gender and personal history. TLM and PBF were the indicators used in the study and were measured by BIA (InBody720, Biospace, Inc., Cerritos, CA, USA), an effective and validated method that was widely used for assessing body composition. BMI was measured by trained investigators using a general formula that the weight divided by the square of the height (kg/m^2^). Biochemistry laboratory data were collected by drawing blood samples from subjects after fasting for at least 8 hours and analyzed by different standard procedures. TC and AST were measured by an enzymatic colorimetric method. The latex-enhanced nephelometry was used to detect hsCRP. UA was measured by the Hitachi 737 automated multichannel chemistry analyzer (Boehringer Mannheim Diagnostics, Indianapolis, IN, USA). Cr was measured by the uncompensated Jaffe method with the alkaline picrate kinetic test. TSH was accessed by an immune-enzymatic assay.

### Statistical analysis

First, odd ratios (ORs) for associations between TLM and PBF with the presence of FBC were performed. A univariate logistic model was applied in the first step (Model 1: unadjusted.) After adjusting pertinent confounders, multivariate models were investigated (Model 2: Model 1 + age, BMI, proteinuria, TC, UA, Cr, AST, albumin, hsCRP, and TSH. Model 3: Model 2 + history of cigarette smoking and alcoholic consumption). Second, multivariable logistic regression was used for PBF predicting the risk of developing FBC with or without the presence of MetS, DM and fatty liver. The effect of modification by FBC and different health outcomes was tested by including interaction terms in the models for the PBF, and the results were shown in the following table. There were significant interactions between FBC with MetS (p < 0.001), DM (p = 0.032) and fatty liver (p = 0.007). According to the significant findings of the interaction testing, further stratified analyses were performed. Finally, ORs for associations between TLM and PBF with the presence of FBC in quartile analysis were conducted. Multivariable models were adjusted as follows. Statistical estimations used in the study were performed by the Statistical Package for the Social Sciences, version18.0 (SPSS Inc., Chicago, IL, USA) for Windows. The differences between males and females in terms of demographic information and biochemistry data were examined by Student’s t test and Pearson’s chi-square test. A two-sided *p*-value of ≤0.05 was regarded as the threshold for statistical significance.
